# Burden of nosocomial COVID-19 in Wales: results from a multicentre retrospective observational study of 2508 hospitalised adults

**DOI:** 10.1136/thoraxjnl-2021-216964

**Published:** 2021-07-22

**Authors:** Mark J Ponsford, Rhys Jefferies, Chris Davies, Daniel Farewell, Ian R Humphreys, Stephen Jolles, Sara Fairbairn, Keir Lewis, Daniel Menzies, Amit Benjamin, Favas Thaivalappil, Chris Williams, Simon M Barry

**Affiliations:** 1 Immunodeficiency Centre for Wales, University of Wales Hospital, Cardiff, UK; 2 Division Infection, Immunity, and Inflammation, School of Medicine, Cardiff University, Cardiff, UK; 3 Respiratory Health Implementation Group, NHS Wales Collaborative, Swansea, UK; 4 Swansea University Medical School, Institute of Life Science, Swansea University, Swansea, UK; 5 The Institute of Clinical Science and Technology, Cardiff, UK; 6 Division of Population Medicine, School of Medicine, Cardiff University, Cardiff, UK; 7 Systems Immunity Research Institute, Cardiff University, Cardiff, UK; 8 Department of Respiratory Medicine, Aneurin Bevan Health Board, Newport, UK; 9 Department of Medicine, Prince Philip Hospital, Hywel Dda University Health Board, Carmarthen, UK; 10 College of Human and Health Sciences, Swansea University, Swansea, West Glamorgan, UK; 11 Department of Respiratory Medicine, Betsi Cadwaladr University Health Board, Bangor, UK; 12 Department of Respiratory Medicine, Cwm Taf University Health Board, Abercynon, UK; 13 Department of Respiratory Medicine, Swansea Bay University Health Board, Port Talbot, UK; 14 Communicable Disease Surveillance Centre, Public Health Wales, Cardiff, UK; 15 Department of Respiratory Medicine, Cardiff and Vale University Health Board, Cardiff, UK

**Keywords:** COVID-19, clinical epidemiology, infection control, viral infection, respiratory Infection

## Abstract

The burden of nosocomial SARS-CoV-2 infection remains poorly defined. We report on the outcomes of 2508 adults with molecularly-confirmed SARS-CoV-2 admitted across 18 major hospitals, representing over 60% of those hospitalised across Wales between 1 March and 1 July 2020. Inpatient mortality for nosocomial infection ranged from 38% to 42%, consistently higher than participants with community-acquired infection (31%–35%) across a range of case definitions. Those with hospital-acquired infection were older and frailer than those infected within the community. Nosocomial diagnosis occurred a median of 30 days following admission (IQR 21–63), suggesting a window for prophylactic or postexposure interventions, alongside enhanced infection control measures.

Little is known regarding the prevalence and outcomes of in-hospital transmission of SARS-CoV-2 among medical patients.^1^ The largest and only multicentre cohort study to date reported outcomes in 1564 patients admitted with confirmed SARS-CoV-2 infection across 11 hospitals.^2^ Mortality in the nosocomial group appeared comparable to those with likely community-acquired infection (27.0% and 27.2%, respectively).^2^ This study was conducted early in the pandemic course, meaning reliable estimates of the true impact of hospital-acquired COVID-19 infection remain hampered by a paucity of publicly available data at national and regional levels.^3^ Here, we update assessment of the relative burden of community-acquired and nosocomial-acquired SARS-CoV-2 infection, using anonymised patient-level and hospital-level data collected via the *National Pathway for Managing COVID-19 Infections in Secondary Care in Wales* initiative (www.covid-19hospitalguideline.wales.nhs.uk).

The methods and data sources relating to this work are described in detail elsewhere.^4^ Briefly, positive SARS-CoV-2 PCR results recorded between 1 March 2020 and 1 July 2020 in adults with a recorded hospital admission were identified for retrospective notes review. Local clinical teams across 18 centres (online supplemental file S1) performed data entry using a standardised online tool. Mandatory fields included dates of PCR sampling, admission and discharge, age, sex, comorbidity count and outcome (death or discharge). Supplementary fields included Welsh Index of Multiple of Deprivation (WIMD) and preadmission Clinical Frailty Scale (CFS).

10.1136/thoraxjnl-2021-216964.supp1 Supplementary data

The primary outcome was all-cause mortality, grouped by probable origin of SARS-CoV-2 infection based on (1) clinician-recorded source and (2) standardised case definitions (online supplemental file S2). Time-to-event analysis used time in hospital following a positive PCR test, to avoid introducing survivorship bias. All analyses were performed using R and GraphPad Prism.

We identified 6005 SARS-CoV-2-positive results with a location in hospital, taken between 1 March 2020 and 1 July 2020 inclusive, of which 4112 were individual cases. Clinical information was obtained from 2584/4112 individuals (63%). A total of 76 individuals were excluded due to missing core data fields or initial PCR sampling date exceeding admission period by 31 days (online supplemental file S3). This left 2508 case records, representing approximately 61% of the total adult population hospitalised with COVID-19 within Wales. Admission features are summarised in table 1. The cohort had a median age of 74 years (IQR 62.5–85.5), of whom 54.3% were men and 45.7% were women. Individuals from the most-deprived WIMD quartile were over-represented relative to those in the least deprived quartile (31.2% vs 18.7%, χ^2^ test: p<0.0001).

**Table 1:** Demographics and clinical features at presentation

Variable	Died (%)	Discharged (%)	Total (%)
Admission hospital			2508
A	174 (40.6)	255 (59.4)	429 (17.1)
B	165 (38.9)	259 (61.1)	424 (16.9)
C	96 (39.8)	145 (60.2)	241 (9.6)
D	78 (32.9)	159 (67.1)	237 (9.4)
E	97 (42.9)	129 (57.1)	226 (9.0)
F	46 (27.1)	124 (72.9)	170 (6.8)
G	35 (22.4)	121 (77.6)	156 (6.2)
H	48 (33.3)	96 (66.7)	144 (5.7)
I	19 (22.6)	65 (77.4)	84 (3.3)
J	22 (27.2)	59 (72.8)	81 (3.2)
K	35 (43.2)	46 (56.8)	81 (3.2)
L	24 (32.0)	51 (68.0)	75 (3.0)
M	14 (21.2)	52 (78.8)	66 (2.6)
N	24 (38.7)	38 (61.3)	62 (2.5)
O	6 (25.0)	18 (75.0)	24 (1.0)
P*	2 (25.0)	6 (75.0)	8 (0.3)
Age group (years)			
<65	115 (14.7)	667 (85.3)	782 (31.2)
65–75	305 (44.4)	382 (55.6)	687 (27.4)
75–85	273 (50.6)	267 (49.4)	540 (21.5)
>85	192 (38.5)	307 (61.5)	499 (19.9)
Sex			
Female	377 (32.9)	768 (67.1)	1145 (45.7)
Male	508 (37.3)	855 (62.7)	1363 (54.3)
Median comorbidity count (IQR)	3 (2–4)	2 (0.5–3.5)	2 (0.5–3.5)
Supplementary fields			
WIMD†			
Q1—most deprived	265 (33.9)	517 (66.2)	782 (31.2)
Q2	251 (38.0)	409 (62.0)	660 (26.3)
Q3	163 (34.5)	310 (65.5)	473 (18.9)
Q4—least deprived	168 (35.7)	302 (64.3)	470 (18.7)
WIMD unrecorded	38 (30.9)	85 (69.1)	123 (4.9)
CFS score			
1—very fit	21 (12.8)	143 (87.2)	164 (6.5)
2—fit	32 (16.1)	167 (83.9)	199 (7.9)
3—managing well	47 (27.3)	125 (72.7)	172 (6.9)
4—vulnerable	63 (39.9)	95 (60.1)	158 (6.3)
5—mildly frail	70 (52.6)	63 (47.4)	133 (5.3)
6—frail	117 (49.6)	119 (50.4)	236 (9.4)
7—severely frail	87 (50.0)	87 (50.0)	174 (6.9)
8—very severely frail	36 (62.1)	22 (37.9)	58 (2.3)
9—terminally ill	7 (63.6)	4 (36.4)	11 (0.4)
CFS score unrecorded	405 (33.7)	798 (66.3)	1203 (48.0)
Ceiling of care			
Intensive care	102 (30.0)	238 (70.0)	340 (13.6)
Ward (CPAP)	98 (42.2)	134 (57.8)	232 (9.2)
Ward (no CPAP)	572 (41.2)	817 (58.8)	1389 (55.4)
Ceiling of care unrecorded	113 (20.7)	434 (79.3)	547 (21.8)

*Represents three combined centres (<5 patients each).

†WIMD, 1=most deprived, 1909=least deprived.

CFS, Clinical Frailty Scale; CPAP, continuous positive airway pressure; WIMD, Welsh Index of Multiple Deprivation.

Clinician-defined admission source was available in 2354 cases (93.9%). Hospital-acquired COVID-19 was documented in 433 cases (17.3% of cohort, 37.8% mortality), comparable to mortality in cases presenting by ambulance (553/1359, 40.7%). Walk-in and GP referrals together accounted for 20.4% of the cohort and had the lowest inpatient mortality rate (17.1%–23.6%). The small number of patients admitted from care or nursing homes showed the highest inpatient mortality rate (23/50, 46.0%).

We next applied a standardised definition for nosocomial COVID-19, based on the interval between admission and diagnostic testing exceeding 14 days, identifying 411 cases (16.4% of cohort, consistent with previous reports).^2^ Community-acquired cases constituted the majority (n=1604, 64.0%), defined by PCR sampling preceding or within 5 days of admission.^2^ Monthly prevalence estimates are shown in online supplemental file S4. Overall, 39.2% of patients with nosocomial-infection died, compared with 31.7% with community-acquired infection. This proved consistent across the majority of admission sites (figure 1). Using a random effects model, we found that the relative risk of mortality in patients with nosocomial-acquired COVID-19, averaged across sites, was 1.24 times that of community-acquired infection (95% CI 1.06 to 1.42, p=0.0047; online supplemental file S5). The median time from diagnostic sampling to discharge in patients with nosocomial infection was 17 days (IQR 7–38), compared with 7 days (IQR 3–15) in community-acquired COVID-19 cases. Nosocomial-infection cases had an increased cumulative incidence of inpatient mortality, when accounting for the competing risk of discharge (figure 2). Half of those with a nosocomial diagnosis had been admitted for at least 30 days prior to testing (IQR 21–63), with 48 admitted for over 100 days. Mortality by age group appeared similar for both nosocomial and community COVID-19 cases, but with a greater proportion of elderly individuals within the nosocomial COVID-19 group (online supplemental file S6).

**Figure 1:**
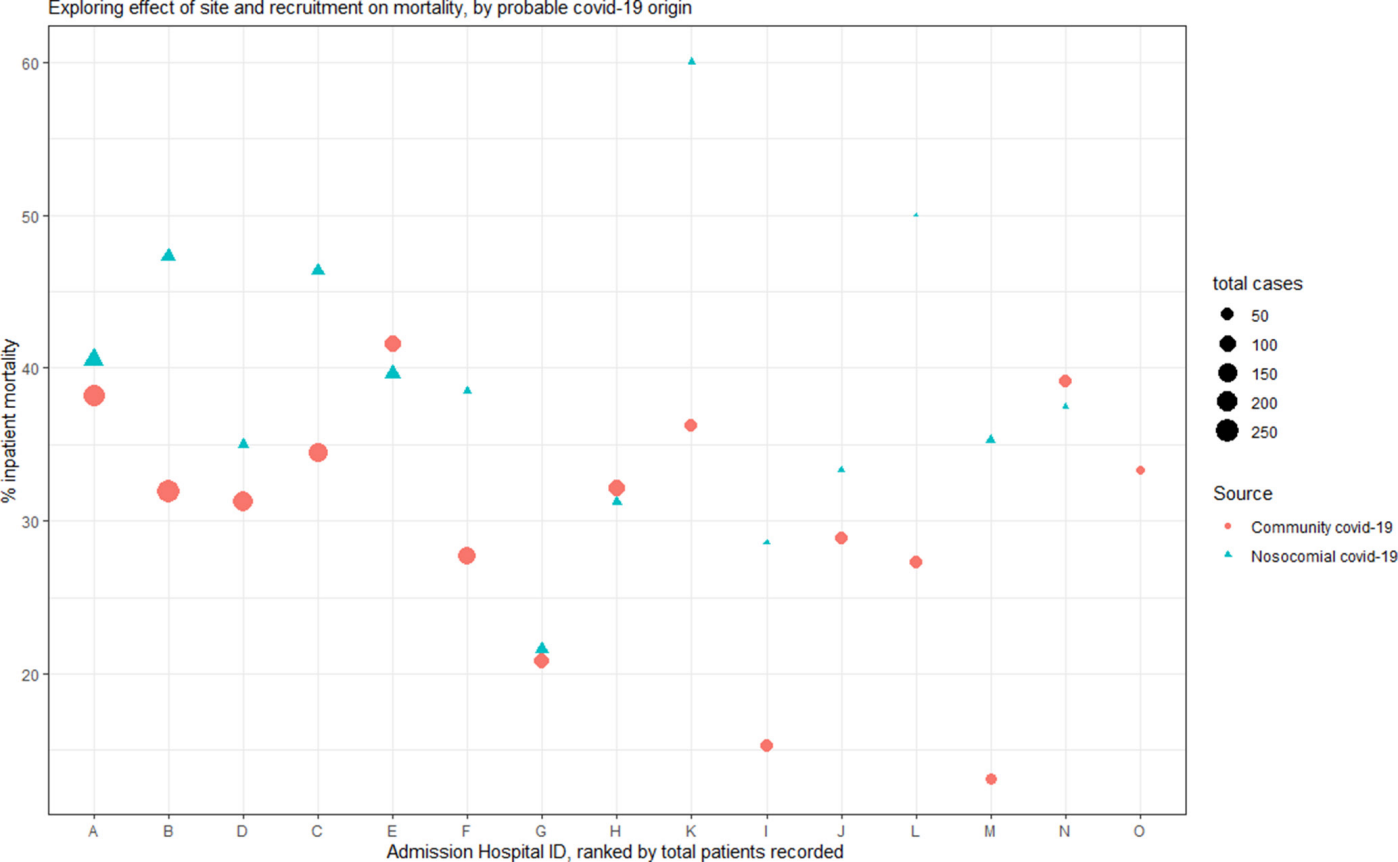
Inpatient mortality rates by admission hospital sites scatter plot showing inpatient mortality rates for patients with community-acquired COVID-19 (circles) and nosocomial COVID-19 (triangles) by individual sites, with hospitals arranged by decreasing overall case load are plotted from the left. for 11/15 sites, inpatient mortality rates for nosocomial cases exceeds that of community acquired cases. Individual sites with fewer than five cases were excluded from analysis.

**Figure 2:**
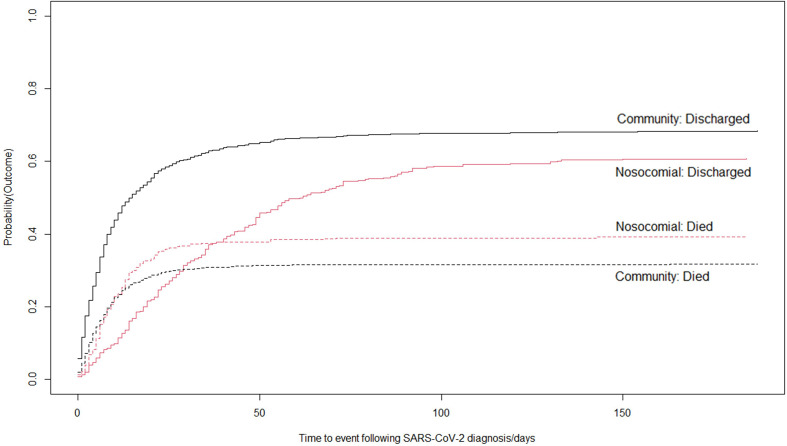
Competing risk analysis plot of nosocomial and community infection outcomes of patients with COVID-19. Time to event analysis cumulative incidence analysis for the competing risks of discharge and diagnosis, using the time from SARS-CoV-2 diagnosis. COVID-19 origin is assigned by the commonly used case definition, as outlined by Carter et al,^2^ nosocomial and community-acquired COVID-19 as labelled. Dotted lines: cumulative incidence of death, continuous lines: cumulative incidence of discharge on probability scale. To deal with potential survivorship bias introduced by including community-diagnoses tested prior to admission (who cannot reach discharge or death until admission), day 0 was defined as the more recent of day of admission or date of first positive diagnostic SARS-CoV-2 testing.

As 95% of individuals display symptoms between 2.5 and 11.5 days of exposure,^5^ the above definition presents a conservative estimate of the burden of nosocomial infection. We extended analysis by varying the diagnostic interval across this range, thereby encompassing wider case definitions in use by UK Public Health agencies. Inpatient mortality rates for nosocomial COVID-19 ranged from 37.8% to 42.3% and remained greater than that for community-acquired infection (31.4%–34.7%, online supplemental files S7 and S8). By contrast, varying the case definition resulted in significant changes in nosocomial caseload and deaths. Applying the Public Health England definition (diagnosis>7 days following admission) identified 7247 (28.9%) admissions and 300 deaths (41.4% mortality). This rose to 827 cases (33.0%) and 341 deaths (41.2% mortality), when a 5-day interval between PCR testing and admission was used.^6^

Finally, we investigated the vulnerability of individuals with nosocomial COVID-19. To minimise selection bias inherent with previous case definitions requiring a prolonged preinfection admission,^2^ we considered a diagnostic interval of 2 days postadmission, commonly used for hospital-acquired bacterial pneumonia.^7^ Here, nosocomial-acquired cases had a median CFS score of 5 (IQR 4–7), compared with 3 (IQR 2–6) in community-acquired cases (online supplemental file S9). Marked differences in multimorbidity were evident, with 35.0% of nosocomial cases having at least four comorbidities compared with 26.7% of community-acquired patients.

Our findings expose the hitherto underestimated vulnerability and impact of nosocomial infection with SARS-CoV-2. Many potential mechanisms may underlie these observations, including the advanced age and frailty of patients who remain admitted to the hospital during the pandemic.^2 8^ These both predispose to severe disease^9^ and implicate personal care requirement as a causal link to exposure.^10^

Study strengths include the high proportion of patients hospitalised with COVID-19 (over 60%) across Wales with available core data, comparing favourably to similar reports.^2 9^ We employed simple but robust statistical methodology, acknowledging the competing risks of discharge and death and multiple case definitions. This is relevant to interpretation of publicly reported figures. For instance, defining nosocomial cases based on the median 5-day incubation period^5^ identified 14.2% additional cases and 13.7% more deaths than a commonly used 7-day threshold. This suggests the burden of nosocomial COVID-19 may be significantly under-reported, which has major public health implications for infection control policy globally, particularly given the rapid spread of more infectious and severe SARS-CoV-2 variants.

Our study also has limitations, including its retrospective nature. Although sites retrieved notes at random, we cannot fully exclude risk of ascertainment bias. As the total number of patients at risk of infection was unknown, we cannot infer the risk of acquiring SARS-CoV-2 within the hospital. Similarly, we did not collect data on recent hospitalisations, and it is possible that nosocomial COVID-19 cases have been classified as community. We also recognise our findings represent crude inpatient mortality rate estimates, based on all-cause mortality. Future studies using national linked datasets including genomic analysis and estimating excess mortality are suggested.

In conclusion, we performed a national service evaluation to document the burden of nosocomial-SARS-CoV-2 infection during and following the first wave in Wales. We found many of those dying with probable hospital-acquired COVID-19 had been in the hospital for at least a month prior to exposure. We suggest this highlights an opportunity for pre-exposure and early postexposure prophylactic measures, including inpatient vaccination and clinical trial enrolment.^11 12^

This work was presented to the Welsh Technical Advisory Group and Directors of Nursing Group, contributing to a recommendation to ministers supporting vaccination of inpatients without a diagnosis of COVID-19 within priority groups and those being admitted for a planned procedure at increased risk.
