# Supplementary material for: Burden of nosocomial COVID-19 in Wales: results from a multicentre retrospective observational study of 2508 hospitalised adults
Source: Thorax. 2021 Jul 22;76(12):1246–9. doi: 10.1136/thoraxjnl-2021-216964 (PMC8606436; doi:10.1136/thoraxjnl-2021-216964)
Supplement: Supplementary data [file thoraxjnl-2021-216964supp001.pdf]

Supplementary material to "The burden of nosocomial covid-19 in Wales: results from a multi-centre retrospective observational study of 2508 hospitalised adults."

Table of Contents

Supplementary S1: List of participating centres 2

Supplementary S2: Commonly used case definitions for probable covid-19 origin. 3

Supplementary S3: Study flowchart 4

Supplementary S4: Monthly prevalence of nosocomial infection diagnosis 5

Supplementary S5: Random effects model for relative risk of mortality by covid-19 origin and site 6

Supplementary S6: Mortality by age-group and covid-19 infection source 7

Supplementary S7: Effect of varying diagnostic cut-off on burden of nosocomial infection . 8

Supplementary S8: Effect of varying case definitions on prevalence and crude mortality rate for community- and nosocomial- SARS-CoV-2 infection 9

Supplementary S9: Comparison of community-acquired and nosocomial-acquired COVID-19 patient characteristics, based on diagnostic interval of 2 days between admission and diagnostic testing 10

Supplementary S1: List of participating centres

Aneurin Bevan Health Board	Nevill Hall Hospital, Royal Gwent Hospital and Ysbyty Ystrad Fawr.
Betsi Cadwalladr Health Board	Glan Clwyd Hospital, Wrexham Maelor Hospital and Ysbyty Gwynedd (Bangor).
Cardiff and Vale Health Board	University Hospital Llandough and University Hospital of Wales.
Cwm Taf Health Board	Prince Charles Hospital, Princess of Wales Hospital and Royal Glamorgan Hospital.
Hywel Dda Health Board	Bronglais Hospital, Glangwili General Hospital, Prince Phillip Hospital and Withybush General Hospital.
Swansea Bay Health Board	Morriston Hospital, Singleton Hospital and Neath Port Talbot Hospital.

All hospitals delivered urgent and emergency care to patients diagnosed with covid-19.

Supplementary S2: Commonly used case definitions for probable covid-19 origin.

Date of first positive SARS-CoV-2 PCR-testing used in all cases.

	**Case definition**	**Probable covid-19 origin**
COPE study investigators [1]	Positive SARS-CoV-2 test taken prior to or within first 5 days of admission	Community-acquired covid-19 ("CAC")
	Positive SARS-CoV-2 test taken between 5-14 days after admission	Probable Community-acquired
	Positive SARS-CoV-2 test taken more than 14 days after hospital admission (*patient required to remain an inpatient on date of swab sampling*)	Hospital-acquired (Nosocomial-"NC")
Public Health England	Positive SARS-CoV-2 test taken after 7 days of hospital admission	Probable Nosocomial covid-19
Public Health Wales	Positive SARS-CoV-2 test taken and no hospital admission within 28 days or within 2 days of hospital admission	Community onset
	Positive SARS-CoV-2 test taken more than 2 days and less than 8 days from hospital admission	Indeterminate hospital onset
	Positive SARS-CoV-2 test taken more than 7 days and less than 15 days from hospital admission	Probable hospital onset
	Positive SARS-CoV-2 test taken more than 14 days from hospital admission	Definite hospital onset

[1] Carter B, Collins JT, Barlow-Pay F, et al. Nosocomial COVID-19 infection: examining the risk of mortality. The COPE-Nosocomial Study (COVID in Older PEople). *Journal of Hospital Infection* 2020; **106**(2): 376-84.

Supplementary S3: Study flowchart

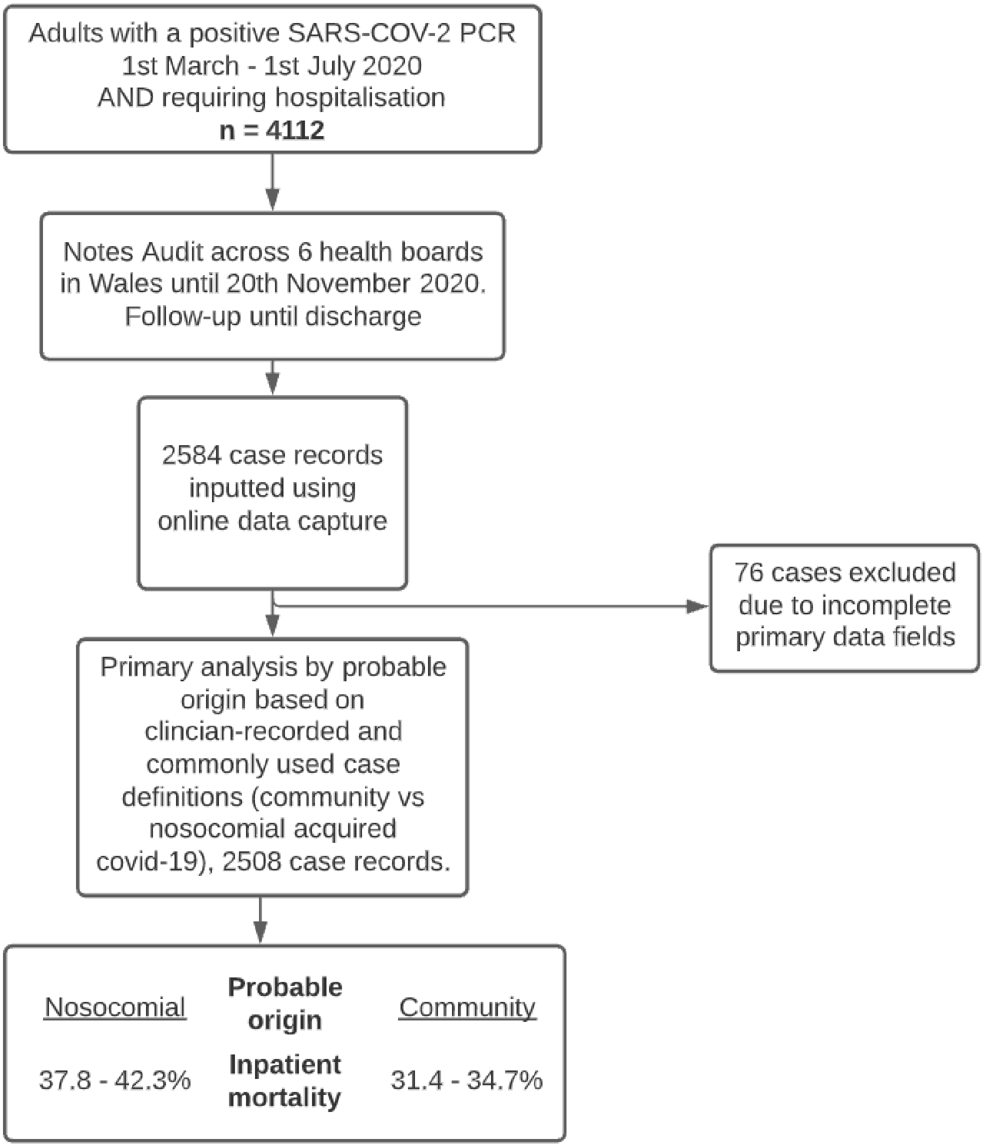

Supplementary S4: Monthly prevalence of nosocomial infection diagnosis

**Month (2020)**	**Total recorded cases**	**COPE/ Public Health Wales case definition***	**Clinician-recoded diagnosis**
		‘Definite’ nosocomial (%)	Hospital-acquired (%)
March	677	96 (14.2%)	117 (17.3%)
April	1348	215 (15.9%)	224 (16.6%)
May	346	73 (21.1%)	67 (19.3%)
June	135	25 (18.5%)	26 (19.3%)

**Defined by diagnostic PCR testing performed >14 days following hospital admission.*

Supplementary S5: Random effects model for relative risk of mortality by covid-19 origin and site

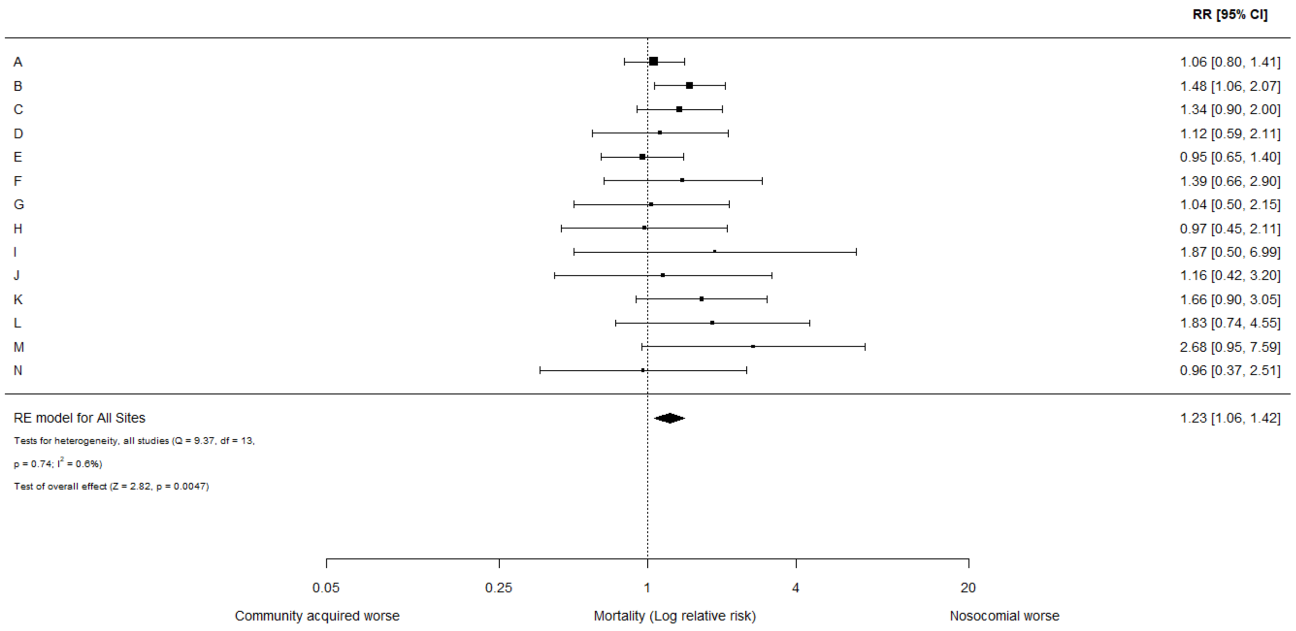

Forest plot assessing the relative risk (RR) and 95% confidence interval (95% CI) of mortality in adults hospitalised with community-acquired and nosocomial covid-19, based on the COPE study definitions[1]. The size of each box is proportional to the size of the individual hospital site (A-N), with the error bars representing the 95% CIs. The diamond represents the pooled average across sites, based on a random effects (RE) model. Small sites not reporting mortality in both patient groups were excluded from analysis. I^2 : heterogeneity variance, calculated using random effects maximum likelihood (REML) and metafor package in R.

Supplementary S6: Mortality by age-group and covid-19 infection source

Age group (years)	Probable covid-19 origin*	Death	Discharged	Mortality (%)	Total cases
<65	Community	91	564	13.9	655
<65	Nosocomial	7	44	13.7	51
65-74	Community	124	197	38.6	321
65-74	Nosocomial	36	56	39.1	92
75-84	Community	175	211	45.3	386
75-84	Nosocomial	56	73	43.4	129
85+	Community	118	14	48.8	242
85+	Nosocomial	62	77	44.6	139

Probable origin as defined by COPE study investigators, see S2.

Supplementary S7: Effect of varying diagnostic cut-off on burden of nosocomial infection

Sensitivity analysis to explore the effect of theoretical and current case definitions across the reported incubation period of SARS-CoV-2 virus. Diagnostic cut-off refers to the number of days elapsed between admission and a positive SARS-CoV-2 test being taken. An interval greater than this threshold is used to define nosocomial covid-19, and an interval less than the threshold community-acquired covid-19.

DIAGNOSTIC CUT-OFF	PROBABLE ORIGIN	DIED	DISCHARGED	TOTAL	MORTALITY (%)
**>2**	Nosocomial	407	591	998	40.8
**≤2**	Community	478	1032	1510	31.7
**>3**	Nosocomial	391	554	945	41.4
**≤3**	Community	494	1069	1563	31.6
**>4**	Nosocomial	377	515	892	42.3
**≤4**	Community	508	1108	1616	31.4
**>5**	Nosocomial	341	486	827	41.2
**≤5**	Community	544	1137	1681	32.4
**>6**	Nosocomial	317	451	768	41.3
**≤6**	Community	568	1172	1740	32.6
**>7**	Nosocomial	300	424	724	41.4
**≤7**	Community	585	1199	1784	32.8
**>8**	Nosocomial	279	392	671	41.6
**≤8**	Community	606	1231	1837	33.0
**>9**	Nosocomial	251	366	617	40.7
**≤9**	Community	634	1257	1891	33.5
**>10**	Nosocomial	228	346	574	39.7
**≤10**	Community	657	1277	1934	34.0
**>11**	Nosocomial	207	328	535	38.7
**≤11**	Community	678	1295	1973	34.4
**>12**	Nosocomial	186	306	492	37.8
**≤12**	Community	699	1317	2016	34.7
**>13**	Nosocomial	173	282	455	38.0
**≤13**	Community	712	1341	2053	34.7
**>14**	Nosocomial	168	263	431	39.0
**≤14**	Community	717	1360	2077	34.5
**>15**	Nosocomial	164	257	421	39.0
**≤15**	Community	721	1366	2087	34.5

Supplementary S8: Effect of varying case definitions on prevalence and crude mortality rate for community- and nosocomial- SARS-CoV-2 infection.

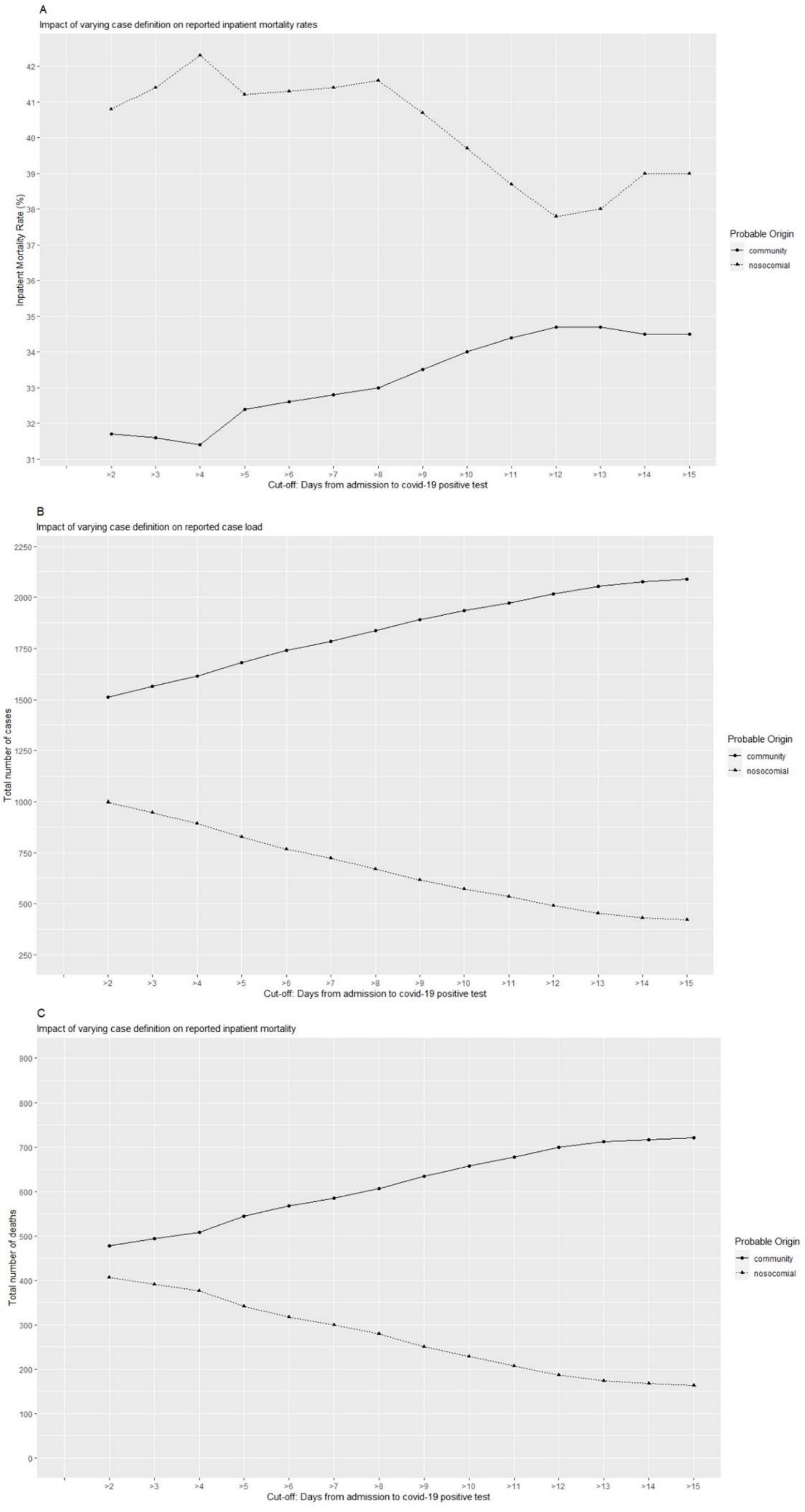

Sensitivity analyses considering the effect on inpatient mortality rate (A), total case numbers (B), and overall mortality burden (C), by varying the case definition across the incubation period, taking admission as the earliest potential nosocomial exposure.

Supplementary S9: Comparison of community-acquired and nosocomial-acquired COVID-19 patient characteristics, based on diagnostic interval of 2 days between admission and diagnostic testing

**Variable, median (IQR)**	**Diagnosed prior to 2-days post admission "Community-acquired"**	**Diagnosed after first 48 hours of admission "Nosocomial-acquired"**	**Univariate Significance**
N	1510 (60.2%)	998 (39.8%)	-
Female (n, %)	661(43.8%)	484(48.5%)	0.0224
Age, years	69 (56-80)	79 (71-87)	<0.0001
Total co-morbidities count	2.0 (1.0-4.0)	3.0 (2.0-4.0)	<0.0001
Clinical Frailty Scale	3 (2-6)<br>*Data available in 862 cases (57%)*	5 (4-7)<br>*Data available in 443 cases (44%)*	<0.0001
Welsh index of multiple deprivation	745 (374-1293)<br>*Data available in 1438 cases (95%)*	772 (403-1310)<br>*Data available in 947 cases (95%)*	0.217
